# The cannabinoid receptors system in horses: Tissue distribution and cellular identification in skin

**DOI:** 10.1111/jvim.16467

**Published:** 2022-07-08

**Authors:** Piotr Kupczyk, Marta Rykala, Pawel Serek, Aleksandra Pawlak, Bartosz Slowikowski, Marcin Holysz, Grzegorz Chodaczek, Jan P. Madej, Piotr Ziolkowski, Artur Niedzwiedz

**Affiliations:** ^1^ Division of General and Experimental Pathology, Department of Clinical and Experimental Pathology, Faculty of Medicine Wroclaw Medical University Wroclaw Poland; ^2^ Department of Internal Medicine and Clinic for Horses, Dogs and Cats, Faculty of Veterinary Medicine University of Environmental and Life Sciences Wroclaw Poland; ^3^ Department of Biochemistry and Immunochemistry, Faculty of Medicine Wroclaw Medical University Wroclaw Poland; ^4^ Department of Pharmacology and Toxicology, Faculty of Veterinary Medicine University of Environmental and Life Sciences Wroclaw Poland; ^5^ Department of Biochemistry and Molecular Biology, Faculty of Medicine Karol Marcinkowski Poznan University of Medical Sciences Poznan Poland; ^6^ Bioimaging Laboratory Lukasiewicz Research Network – PORT Polish Center for Technology Development Wroclaw Poland; ^7^ Department of Immunology, Pathophysiology and Veterinary Preventive Medicine, Faculty of Veterinary Medicine Wroclaw University of Environmental and Life Sciences Wroclaw Poland

**Keywords:** cannabinoid receptors, dermis, epidermis, fibroblasts, keratinocytes, PGP 9.5

## Abstract

**Background:**

The endocannabinoid system (ECS) is composed of cannabinoid receptors type 1 (CBR1) and type 2 (CBR2), cannabinoid‐based ligands (endogenous chemically synthesized phytocannabinoids), and endogenous enzymes controlling their concentrations. Cannabinoid receptors (CBRs) have been identified in invertebrates and in almost all vertebrate species in the central and peripheral nervous system as well as in immune cells, where they control neuroimmune homeostasis. In humans, rodents, dogs, and cats, CBRs expression has been confirmed in the skin, and their expression and tissue distribution become disordered in pathological conditions. Cannabinoid receptors may be a possible therapeutic target in skin diseases.

**Objectives:**

To characterize the distribution and cellular expression of CBRs in the skin of horses under normal conditions.

**Animals:**

Fifteen healthy horses.

**Methods:**

Using full‐thickness skin punch biopsy samples, skin‐derived primary epidermal keratinocytes and dermal‐derived cells, we performed analysis of *Cnr1* and *Cnr2* genes using real‐time PCR and CBR1 and CBR2 protein expression by confocal microscopy and Western blotting.

**Results:**

Normal equine skin, including equine epidermal keratinocytes and dermal fibroblast‐like cells, all exhibited constant gene and protein expression of CBRs.

**Conclusions and Clinical Importance:**

Our results represent a starting point for developing and translating new veterinary medicine‐based pharmacotherapies using ECS as a possible target.

**Abbreviations**
2‐AG: 2‐arachidonoylglycerol
5‐HT1: serotonin 1A receptor or 5‐HT1A receptor
BBB: blood‐brain barrier
CBD: cannabidiol
CBR1: cannabinoid receptors type 1
CBR2: cannabinoid receptors type 2
CBRs: cannabinoid receptors
CNS: central nervous system
DRG: dorsal root ganglion
ECS: endocannabinoid system
GPCRs: G protein‐coupled receptors
GPR55: G protein‐coupled receptor 55
Pan‐CK: pan‐cytokeratin
PEA: palmitoylethanolamide
PGP 9.5: protein gene product 9.5
PNS: peripheral nervous system
PPAR‐α: peroxisome proliferator‐activated receptor alpha
PPAR‐γ: peroxisome proliferator‐activated receptor gamma
TRPA1: transient receptor potential A member 1
TRPV1: transient receptor potential cation channel subfamily V member 1
Δ9‐THC: (−)‐Δ9‐tetrahydrocannabinol

## 1 INTRODUCTION

The endocannabinoid system (ECS) includes cannabinoid receptors (CBRs) and their ligands.^1^ The CBRs are represented by cannabinoid receptor type 1 (CBR1) and type 2 (CBR2), members of the rhodopsin‐like superfamily of 7‐transmembrane G protein‐coupled receptors (GPCRs).^2^, ^3^ They are evolutionarily conserved and have been characterized in all vertebrates and in many invertebrate species.^4^, ^5^ Initially, it was assumed that CBR1 is predominantly distributed within different areas of the central nervous system (CNS), whereas CBR2 dominates on immune cells.^6^ However, it is currently known that CBRs are present in both locations as well as in peripheral tissues, where their balanced expression regulates the neuroimmunological homeostasis of multiple tissues.^7^ The CBRs activation and signal transduction pathways are initiated after binding of 3 general ligand subtypes.^8^ First, naturally‐synthesized endocannabinoids are represented by anandamide (N‐arachidonoylethanolamine) and 2‐arachidonoylglycerol (2‐AG), which bind classical CBRs. In turn, another endogenous ligand, palmitoylethanolamide (PEA), has an affinity to non‐CBRs restricted to peroxisome proliferator‐activated receptor alpha (PPAR‐α). Palmitoylethanolamide also may act on G protein‐coupled receptor 55 (GPR55) and transient receptor potential cation channel subfamily V member 1 (TRPV1).^9^, ^10^ Non‐CBRs were identified in experimental studies using CBR1^−/−^ and CBR2^−/−^ knockout mice and are represented by others including TRPV1, GPR55, PPAR‐α, peroxisome proliferator‐activated receptor gamma (PPAR‐γ), transient receptor potential A member 1 (TRPA1), and serotonin 1A receptor or 5‐HT1A receptor (5‐HT1). Exogenous phytocannabinoids, cannabidiol (CBD), and the psychoactive (−)‐Δ9‐tetrahydrocannabinol (Δ9‐THC) are natural compounds of *Cannabis sativa*, a plant used in medicine since ancient times. Finally, cannabinoid‐based compounds deserve the most attention, featuring well‐defined specificity and pharmacokinetics, which partially or entirely inhibit or activate CBRs signaling.^11^

The CBR‐based treatments have been applied successfully in several pathological conditions in humans and in animal disease models.^2^ Further investigation has indicated that several common and chronic pro‐inflammatory skin diseases, such as atopic dermatitis, psoriasis, fibrotic disorders, and skin cancers, can alter the expression of CBRs.^12^, ^13^, ^14^, ^15^ In turn, experimental studies have shown that disordered cutaneous homeostasis might be established in a CBRs‐dependent manner.^13^, ^16^, ^17^ Accordingly, peripheral application of cannabinoid‐related compounds has shown therapeutic benefits, decreasing associated comorbidities and reconstituting proper skin tissue architecture and physiology.^18^, ^19^

These CBR‐based treatments recently have attracted considerable attention in equine veterinary medicine after their use in other vertebrate species, including dogs, cats, and pigs.^20^, ^21^, ^22^, ^23^, ^24^ Unfortunately, knowledge about the ECS in horses is still limited to dorsal root ganglion (DRG) neurons and equine genome sequencing analysis.^25^, ^26^, ^27^, ^28^ No studies on CBRs distribution and cellular expression in the cutaneous milieu are available. We present a detailed and advanced in situ and in vitro characterization of the equine ECS, using skin punch biopsy samples and primary keratinocyte and fibroblast cultures from horses cultures. Real‐time PCR, Western blotting (WB), and confocal microscopy were used to estimate CBR mRNA transcripts and protein concentrations. These preliminary results may suggest strategies for developing and implementing peripheral CBR‐based dermatological treatments for horses.

## 2 MATERIAL AND METHODS

### 2.1 Animals

Skin samples were collected under sterile conditions from the metacarpal area of healthy horses (n = 15). The characteristics of the horses are presented in the Table S1. All laboratory procedures described below are detailed in the Data S1.

### 2.2 Sample collection and tissue processing

Skin samples were collected into stabilizing reagents and appropriate fixatives, depending on the procedure. For gene and protein expression analysis, skin samples were placed in molecular biology reagent (RNA‐stay, A&A Biotechnology, Gdansk, Poland) and frozen at −80°C. For histology, skin biopsy samples were placed in 10% buffered formalin for 48 hours, and formalin‐fixed paraffin‐embedded (FFPE). For frozen sections, biopsy samples were fixed in 4% paraformaldehyde (4% PFA, POCH S.A., Gliwice, Poland) and embedded in OCT medium (Thermo Fisher Scientific, Waltham, Massachusetts, USA) as described previously.^29^ Finally, the skin was placed in culture medium to initiate skin‐derived primary cell expansion. Moreover, the brains from 3 healthy horses were collected as reference material.

### 2.3 Histology

The FFPE 5‐μm paraffin sections were subjected to a standard procedure of deparaffinization. Each sample was stained by hematoxylin and eosin (H&E; Roth GmbH, Karlsruhe, Germany and POCH S.A., Gliwice, Poland).

### 2.4 Confocal microscopy

The OCT 12‐μm‐thick frozen sections were prepared on glass slides (Ultra Superfrost Plus, Thermo Fisher Scientific, Waltham, Massachusetts, USA) and further subjected to an immunofluorescence protocol, with minor modifications.^29^ After post‐fixation and incubation in the blocking solution (BS), sections were incubated with a combination of primary antibodies: rabbit polyclonal anti‐CBR1, rabbit polyclonal anti‐CBR2, mouse monoclonal anti‐pan‐cytokeratin, mouse monoclonal anti‐vimentin, and chicken polyclonal anti‐PGP 9.5 overnight at 4°C. The next day, sections were incubated with goat‐anti‐rabbit DyLight488, goat‐anti‐chicken DyLight594, and goat‐anti‐mouse DyLight633 (all primary and secondary antibodies purchased from Thermo Fisher Scientific, Waltham, Massachusetts, USA) for 2 hours at room temperature (RT) as well as counterstained with DAPI (Santa Cruz Biotechnology, Santa Cruz, California, USA). The protocol described above also was used for in vitro analysis. Fluorescence intensity (FI) analysis was performed as previously described with minor modification using Fiji‐ImageJ Software (Fiji‐ImageJ, National Institute of Health, Bethesda, Maryland, USA).^29^, ^30^

### 2.5 Primary cell cultures

The skin samples were washed in buffer, and epidermis was separated from the dermis mechanically after incubation in dispase II solution (2.4 U/mL, CnT‐DNP‐10, CellnTec, Bern, Switzerland) at 37°C. Subsequently, the epidermis was cut into small pieces and incubated in 0.25% trypsin with 0.05% ethyl‐enediaminetetraacetic acid (EDTA; Trypsin‐EDTA Solution, Sigma Aldrich, Saint Louis, Missouri, USA). Enzymatic activity was stopped using medium containing 10% fetal bovine serum (FBS, Gibco‐Thermo Fisher Scientific, Waltham, Massachusetts, USA). After centrifugation (200*g*, 7 minutes, 4°C), the cell pellet was resuspended in an appropriate culture medium in a 6‐well culture plate with addition 1% of streptomycin/penicillin solution (Sigma Aldrich, Saint Louis, Missouri, USA). The keratinocytes were cultivated in Epidermal Keratinocyte Medium (CnT‐09, CellnTec, Bern, Switzerland) supplemented with CnT‐IsoBoost Supplement (CnT‐ISO‐50, CellnTec, Bern, Switzerland), whereas fibroblasts were cultivated in DMEM medium (Institute of Immunology and Experimental Therapy, Polish Academy of Sciences, Wroclaw, Poland) supplemented with 10% FBS (Gibco‐Thermo Fisher Scientific, Waltham, Massachusetts) and 2 mM of L‐glutamine (Sigma Aldrich, Saint Louis, Missouri, USA) at 37°C and under 5% CO_2_.

### 2.6 RNA isolation, reverse transcription, and real‐time PCR

The total RNA from equine skin biopsy samples as well as primary keratinocytes and fibroblasts was isolated using protocols described previously.^29^ Briefly, skin samples were homogenized in Phenosol Plus (A&A Biotechnology, Gdansk, Poland) and RNA was isolated using the Universal RNA Purification kit (EurX—Molecular Biology Products, Gdansk, Poland) according to the manufacturer's recommendations. Briefly, 0.3 μg of skin tissue and 1 μg of cell RNA were used for cDNA synthesis, using the smaRT First‐Strand cDNA Synthesis Kit (EurX—Molecular Biology Products, Gdansk, Poland) according to the manufacturer's recommendations. The reaction mix (per well) included 5 μL of UPL ProbeMaster (Roche, Bazylea, Switzerland), 0.5 μM of forward and reverse primers (Genomed, Warsaw, Poland), and 0.2 μM of Universal Probe Library (UPL, Roche, Bazylea, Switzerland) and their respective hydrolysis probes. Real‐time PCR was performed using a LightCycler 480 II (Roche Molecular Systems Inc., Indianapolis, Indiana, USA) with the following conditions: pre‐incubation at 95°C for 10 minutes, 50 cycles of amplification: 15 seconds at 95°C for denaturation, 30 seconds at 58°C for annealing, and 10 seconds at 72°C for elongation. All gene expression analyses were performed in triplicates in the 3 independent experiments.

### 2.7 Western blot

All analyzed tissues and cells underwent protein extraction with RIPA lysis buffer (Sigma‐Aldrich, Saint Louis, Missouri, USA) supplemented with protease cocktail inhibitor (Sigma‐Aldrich, Saint Louis, Missouri, USA) for 20 minutes at 4°C. The 20 μg of protein determined by the bicinchoninic acid assay kit (Thermo Fisher Scientific, Waltham, Massachusetts, USA) was subjected to the routine protocol, and membranes were incubated overnight at 4°C with rabbit polyclonal anti‐CBR1 and rabbit polyclonal anti‐CBR2 (both from Thermo Fisher Scientific, Waltham, Massachusetts, USA). For detection, horseradish peroxidase (HRP)‐conjugated goat anti‐rabbit antibodies (Invitrogen, Waltham, Massachusetts, USA) in Phosphate‐buffered Silane‐tween 20 (PBS‐T) buffer were applied, similarly to HRP‐conjugated anti‐β‐actin antibodies (1:5000, Santa Cruz Biotechnology, Santa Cruz, California, USA) used as the reference control. The proteins were analyzed and visualized using the Clarity Western ECL chemiluminescent substrate (Bio‐Rad, Hercules, California, USA) in the ChemiDoc MP Imaging System (Bio‐Rad, Hercules, California, USA). The antibodies used in WB are provided in Table 1.

**TABLE 1:** Primary non‐conjugated and HRP‐conjugated antibodies as well as secondary antibodies fluorochrome‐conjugated for immunofluorescence (IF) microscopy and HRP‐conjugated for Western‐blot (WB) analysis

Antibody	Host, isotype, clonality, conjugation, target	Dilution * (IF, WB)	Catalog number	Company, Country
Primary	Rabbit IgG polyclonal anti‐CBR1	1:250 (IF) 1:1000 (WB)	PA1‐743	Thermo Fisher Scientific, United States
	Rabbit IgG polyclonal anti‐CBR2	1:250 (IF) 1:1000 (WB)	PA1‐744	Thermo Fisher Scientific, United States
	Chicken IgY polyclonal anti‐PGP 9.5	1:500 (IF)	PA1‐10011	Thermo Fisher Scientific, United States
	Mouse IgG1 monoclonal anti‐cytokeratin	1:500 (IF)	MA5‐12135	Thermo Fisher Scientific, United States
	Mouse IgG1 kappa monoclonal anti‐vimentin	1:500 (IF)	MA5‐11883	Thermo Fisher Scientific, United States
	Mouse IgG1 monoclonal anti‐β‐actin‐HRP	1:5000 (WB)	SC‐47778	Santa Cruz Biotechnology, United States
Secondary	Goat IgG polyclonal anti‐rabbit‐DyLight488	1:500 (IF)	#35552	Thermo Fisher Scientific, United States
	Goat IgG polyclonal anti‐chicken‐DyLight594	1:500 (IF)	SA5‐10073	Thermo Fisher Scientific, United States
	Goat IgG polyclonal anti‐mouse‐DyLight633	1:500 (IF)	#35512	Thermo Fisher Scientific, United States
	Goat IgG polyclonal anti‐rabbit HRP	1:25000 (WB)	A27036	Invitrogen, United States

### 2.8 Data presentation and statistical analysis

Statistical analysis was performed by Kruskal‐Wallis a 1‐way analysis of variance (ANOVA) using GraphPad Prism 5.0 (GraphPad Software Inc., La Jolla, California, USA). Figures were prepared using LibreOffice 5.0 Software (The Document Foundation, Berlin, Germany).

## 3 RESULTS

### 3.1 Histological and morphological analyses of skin

**FIGURE 1:**
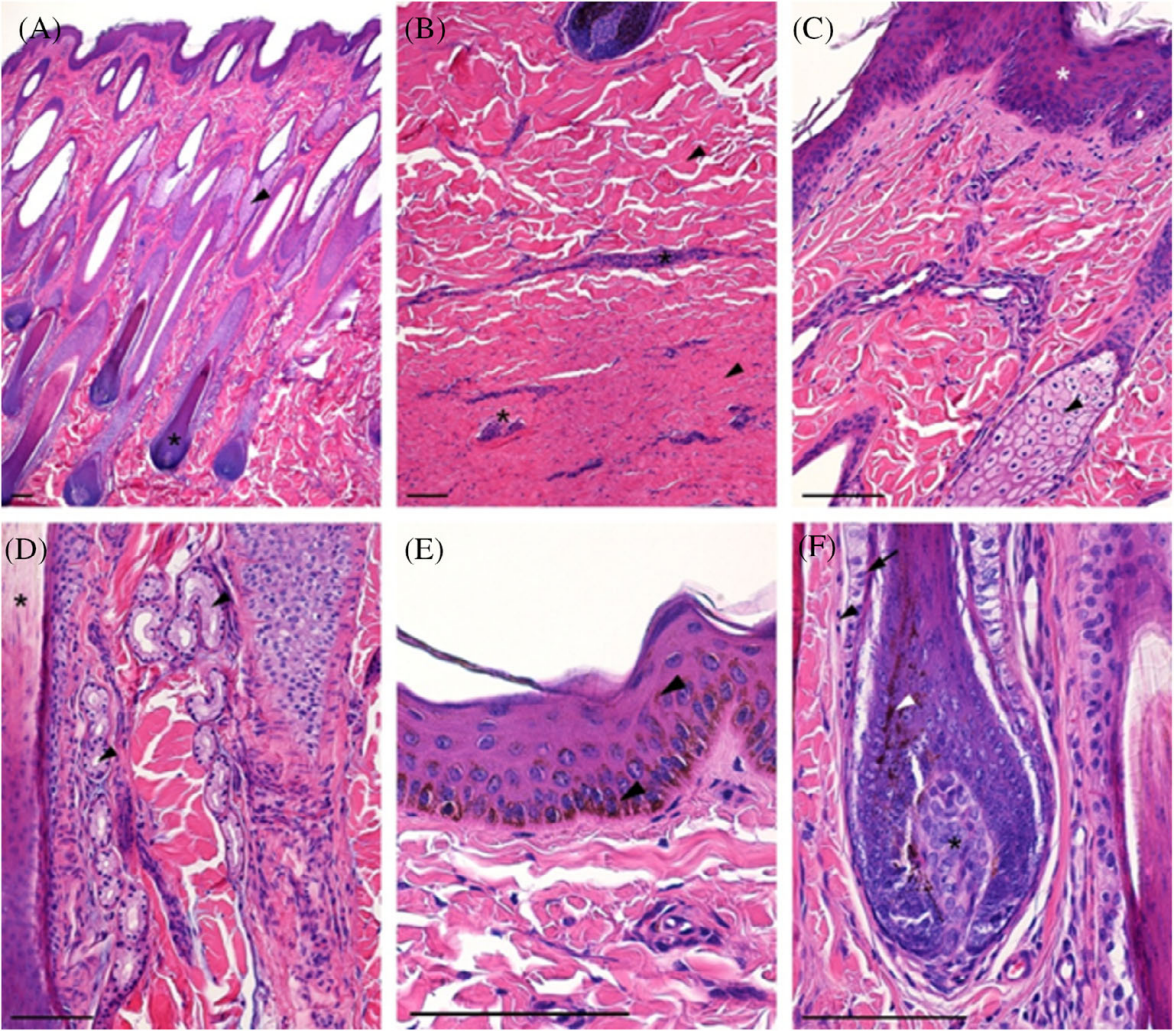
Normal horse skin. (A) Papillary layer of the dermis with hair follicles (asterisk) and accompanying sebaceous glands (arrowhead); (B) Reticular layer of the dermis with thick bundles of collagen fibers (arrowheads) and blood vessels (asterisk); (C) Superficial part of the dermis with epidermis (asterisk) and hair sebaceous gland; (D) Deeper part of the papillary layer with sweat gland (arrowhead) between hair follicles (asterisk); (E) Epidermis (keratinized stratified squamous epithelium) with numerous melanin granules (arrowhead); (F) Hair bulb with pigmented cells (white arrowhead) attached to the dermal papilla (asterisk) and covered by hair follicle with epithelial hair root sheath (arrow) and dermal sheath (arrowhead); H&E staining, scale bar = 100 μm

The skin of the studied horses was normal, without visible histopathological changes. The epidermis consisted of 4 to 7 layers of epithelial cells, covered by an outer keratinized layer (Figure 1).

### 3.2 *In‐situ* distribution and expression analysis of CBRs in whole skin and brain tissue reference material

The CBRs distribution within whole equine skin tissue sections was determined using confocal microscopy. Considering the high expression of CBRs in the brain of other species, the cortex sections were used as reference material, and in the case of both receptors, positive immunoreactivity was confirmed. The CBR1 was present in different sets of neuronal cells and in perivascular regions of microcirculation within respective endothelial cells, in contrast to CBR2 immunoreactivity, in which lower expression was observed (Figure S1).

### 3.3 Epidermal expression of CBRs

**FIGURE 2:**
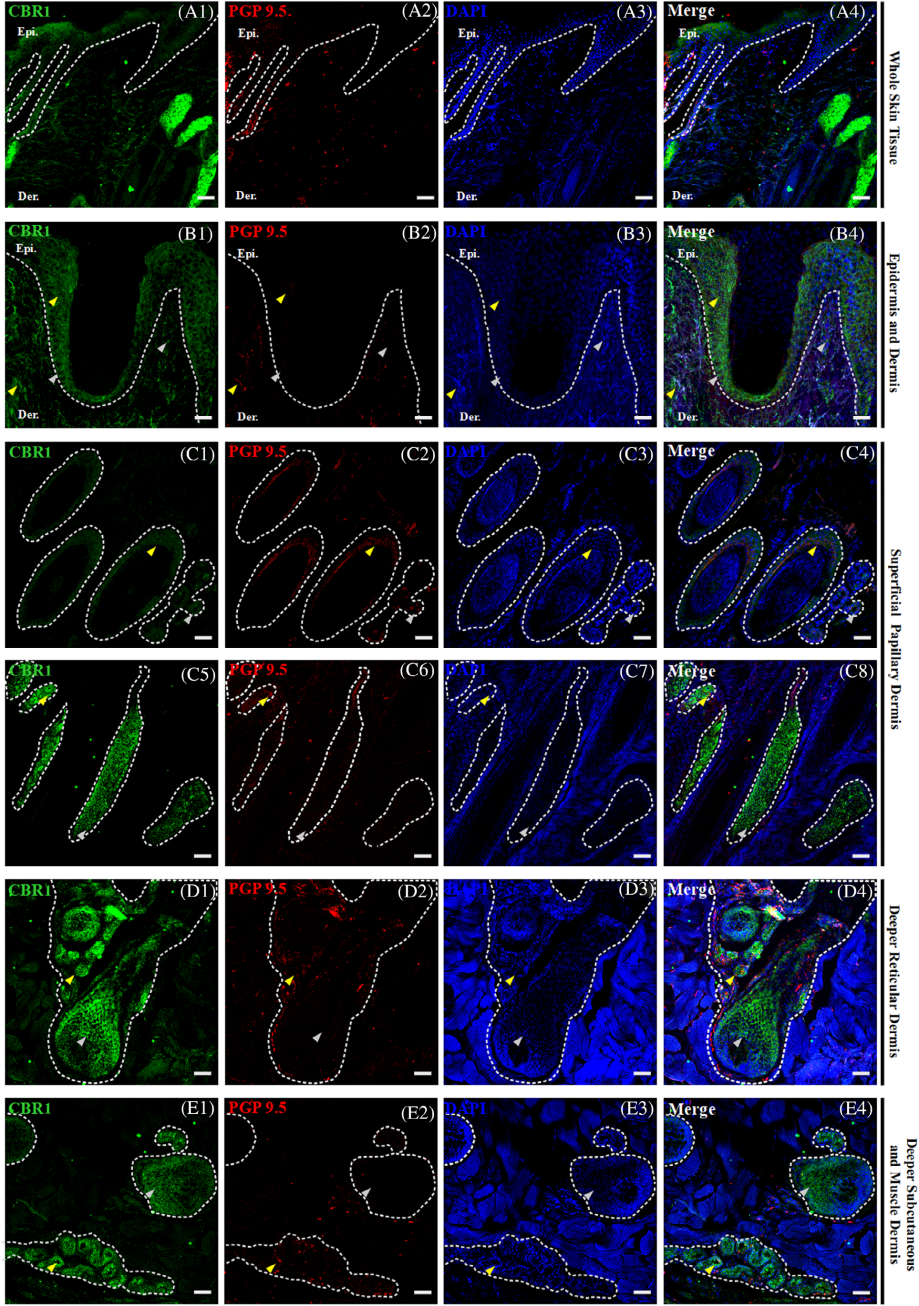
Triple confocal microscopic in‐situ expression of CBR1 (green), PGP 9.5 (red) and DAPI (blue) in the whole skin tissue under 20x magnification; scale bars = 50 μm (A1‐A4). Below, a set of images presenting the layers of the equine skin, with emphasis on their respective epidermal and dermal compartments. Epidermis (Epi.: stratum basale, granulosum, spinosum and corneum; B1‐B2) and dermis (B1‐B2) and dermis (Der.: upper and lower superficial papillary dermis—C1‐C8, deeper reticular dermis—D1‐D4, and deep subcutaneous and muscle dermis—E1‐E4) with their respective compartments. The specific region of interest (white dotted lines) and randomly selected example regions with single CBR1 (gray arrows) and double positive in co‐localization with PGP 9.5 (yellow arrows) specific immunoreactivity are indicated. Images were taken at 40x magnification; scale bars = 20 μm

**FIGURE 3:**
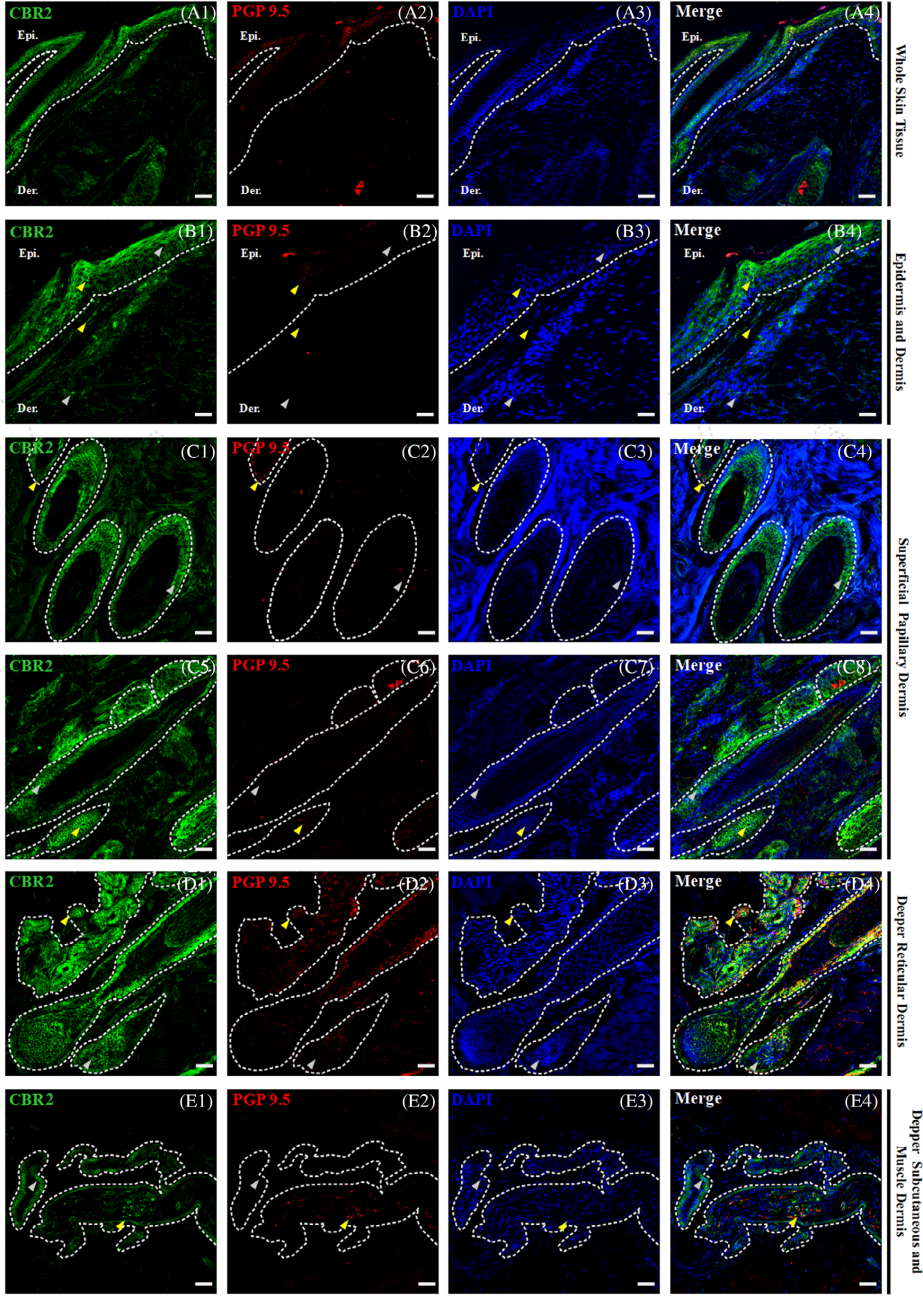
Triple confocal microscopic in‐situ expression of CBR2 (green), PGP 9.5 (red) and DAPI (blue) in the whole‐skin tissue under 20x magnification; scale bars = 50 μm (A1‐A4). Below, a set of images presenting the layers of the equine skin, with emphasis on their respective epidermal and dermal compartments. Epidermis (Epi.: stratum basale, granulosum, spinosum and corneum; B1‐B2) and dermis (B1‐B2) and dermis (Der.: upper and lower superficial papillary dermis—C1‐C8, deeper reticular dermis—D1‐D4, and deep subcutaneous and muscle dermis—E1‐E4) with their respective compartments. The specific region of interest (white dotted lines) and randomly selected example regions with single CBR2 (gray arrows) and double positive in co‐localization with PGP 9.5 (yellow arrows) specific immunoreactivity are indicated. Images were taken under 40x magnification, scale bars = 20 μm

The equine epidermis is a stratified squamous epithelium mainly composed of keratinocytes. Four main compartments are distinguished within the epidermis: strata basale, spinosum, granulosum, and corneum, which, regarding their CBRs expression levels, all show positive and homogenous immunoreactivity with comparable distribution and expression within all examined samples. In all skin compartments, CBRs immunoreactivity was observed in the cytoplasm, whereas signals were rarely observed in the nuclei of single cells in the whole skin tissue (Figures 2 and 3).

The epidermal expression of CBR1 was low, but all epidermal compartments demonstrated positive CBR1 immunoreactivity (Figure 2A‐B4). Of all epidermal compartments, the stratum basale showed the lowest CBR1 expression. In turn, the upper layer of the epidermis showed a slightly higher expression, which was almost identical in the strata spinosum, granulosum, and corneum (Figure 2B1‐B4). In contrast to CBR1, epidermal expression of CBR2 was significantly higher (Figure 3A1‐B4). Although the expression of CBR2 in the stratum basale was similar to that of CBR1, its distribution was more heterogeneous within suprabasilar layers. Stratum spinosum and granulosum and, albeit to a lesser extent, stratum corneum clearly expressed the highest amounts of CBR2. Co‐labeling of CBRs with protein gene product 9.5 (PGP 9.5) provided a weak signal in the stratum basale of the epidermis and by single cells of all suprabasilar epidermal areas (Figure 3B1‐B4).

### 3.4 Dermal expression of CBRs

The equine dermis has a heterogeneous cell population of which fibroblasts are the primary cells supported by skin dendrocytes, principal elements of the equine cutaneous immune system.^31^ The dermis is subdivided into the superficial papillary and deep reticular dermis. In the upper regions of the superficial papillary dermis, just below the epidermis, CBR1 (Figure 2C1‐C4) and CBR2 (Figure 3C1‐C4) expression was seen in fibroblast‐like cells, and their expression levels decreased in the deeper dermis parts. In the lower parts of the superficial dermis, many hair follicles could be observed in cross‐section and some single follicles in the longitudinal hair sections (Figures 2D1‐D4 and 3D1‐D4). Strong CBR2 strong immunoreactivity was found in the hair follicles, where expression levels were homogenous along the structure of the hair, starting from the hair tip, where visible expression was detected along the inner root sheath of the hair in contrast to the outer root sheath, where expression was barely seen and ending on the dermal papilla and hair follicle bulb, where expression was observed (Figure 3C1‐D4). Regarding CBR1, low or even complete absence of CBR1 expression was observed for most hair follicles in the longitudinal and cross‐hair sections in almost all histological compartments (Figure 2C1‐E4). Detection of CBR1 occurred just above the hair follicle bulb formation, in the inner root sheath and the dermal papilla, and the cells surrounding and located under the hair follicle bulb (Figure 2C1‐D4). Here, in the case of both receptors, an intensified signal from PGP 9.5 was observed, and its enhanced expression overlapped with CBR1 in hair areas. Cytoplasmic expression was observed in active basal cells of the inner root sheath, whereas point or linear PGP 9.5 signals corresponded to DRG nerve fibers that terminated in the hair follicle bulb. Several PGP 9.5 fibers also were seen outside the hair follicle bulb, in the surrounding muscle tissues and sebaceous glands (Figure 2D1‐D4). The latter demonstrated the strongest CBR1 expression of all analyzed skin structures, which was especially noticeable within all parts of the superficial papillary dermis, whereas CBR2 showed significantly less but visible expression in sebaceous and sweat glands (Figure 3D1‐D4). In the latter, CBR1 immunoreactivity dominated over CBR2 immunoreactivity, which was observed in the cross‐sections of sweat glands in the deeper reticular dermis and in the deeper subcutaneous dermis and the muscle dermis (Figure 3E1‐E4). Similarly, several point PGP 9.5 signals were observed to co‐localize with CBR1 and CBR2 expression in this skin area. Finally, the lower part of the deeper subcutaneous and muscle dermis layer, where adipocytes dominate, are highly abundant in the microvascular networks where CBR2 immunoreactivity is strong in endothelial cells and their perivascular cells (pericytes, vascular smooth muscle cells, and adventitial cells) (Figure 3E1‐E4) in opposition to CBR1 (Figure 2E1‐E4).

**FIGURE 4:**
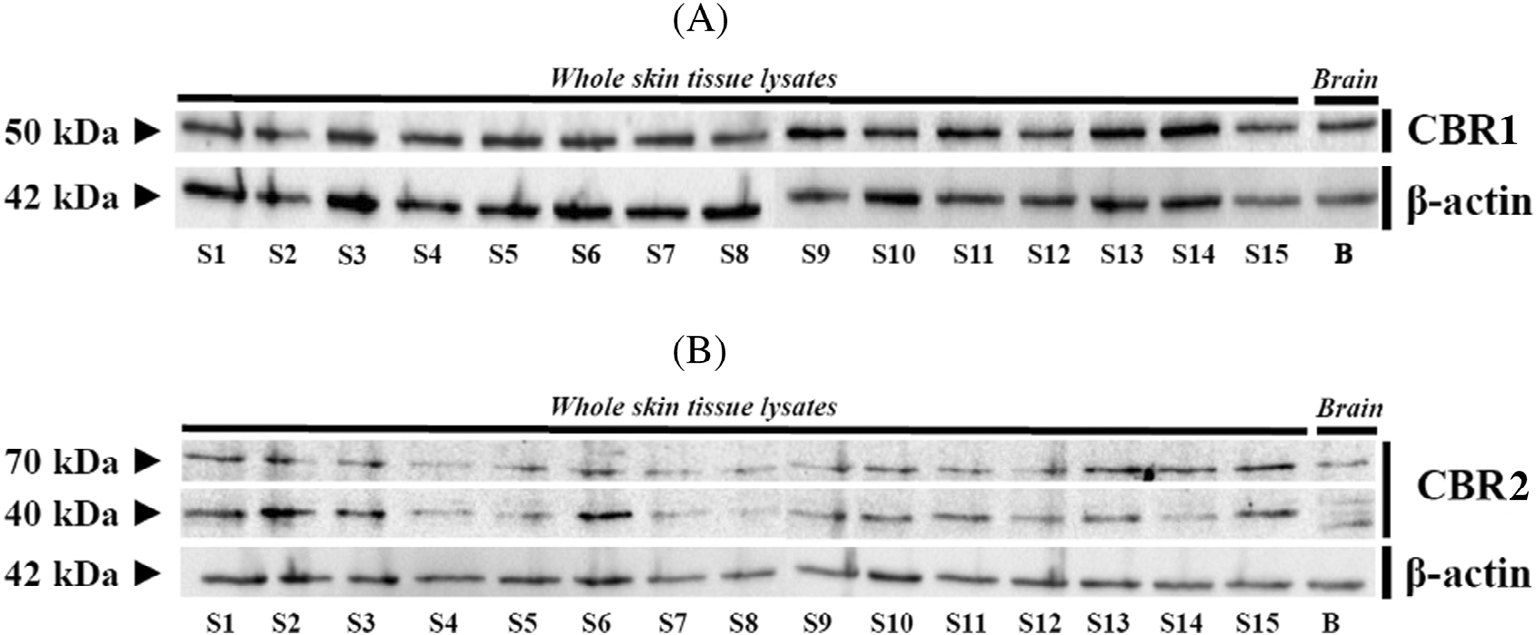
Western‐blot whole skin tissue protein expression for CBR1 (A) and CBR2 (B) in all equine samples and brain reference tissues

However, although rabbit polyclonal antibodies for CBRs, applied during confocal microscopy, allowed us to demonstrate CBRs distribution in the tissues, verification to assess their specificity, as well as quantification of the CBRs, was performed using a WB method in the whole skin tissue and brain samples (Figure 4).

The WB assays for CBR1 showed 1 immunoreactive band in each lane corresponding to equine skin biopsy samples from 15 different individuals, with a molecular mass of 50 kDa (Figure 4A). In the WB assay for CBR2 in skin biopsy samples, 2 bands were detected at approximately 70 and 40 kDa (Figure 4B). The lower band corresponded to a deglycosylation or proteolysis product of the 70 kDa band.^32^ The lane on the right shows the presence of similar bands in samples of equine cerebral cortex protein extract, which was used as a positive control for both CBR1 and CBR2 (Figure 4). The β‐actin immunoreactivity used as a reference protein was correctly observed in whole skin tissue lysates of the investigated group as well as in brain‐derived samples, with a band corresponding to 42 kDa.

### 3.5 *In‐vitro* expression analysis of CBRs in primary skin‐derived keratinocyte and dermal‐derived cell cultures

**FIGURE 5:**
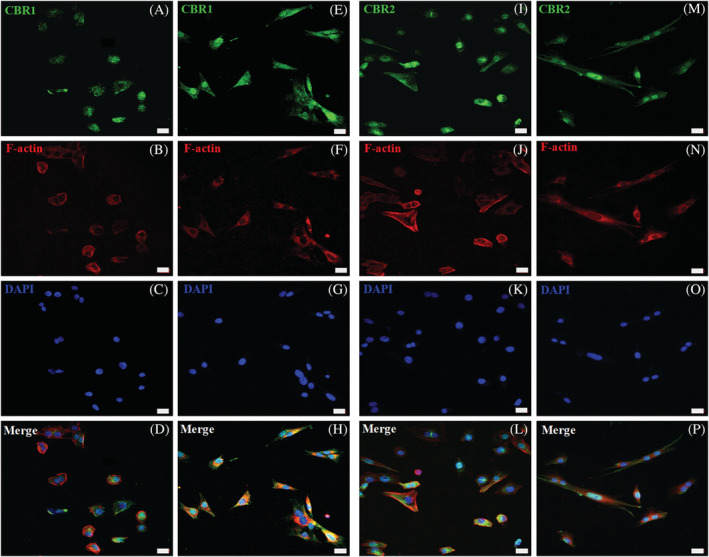
Triple confocal microscopic images representing in‐vitro CBR1 and CBR2 (green) expression in equine primary skin‐derived cells. Immunoreactivity in the primary skin‐derived keratinocytes was detected for CBR1 (A‐D) and CBR2 (I‐L). Similarly, primary skin‐derived dermal cells were, in most present fibroblasts, also positive for CBR1 (E‐H) as well as CBR2 (M‐P). The cell cytoskeletal analysis of F‐actin (Phalloidin‐AF555 staining, red), with its content and distribution, was performed to distinguish keratinocytes (B and J) from fibroblasts (F and N). The DAPI (blue) was used for nucleus counterstaining of keratinocytes (C and K) and fibroblasts (G and O). Images were taken under 40x magnification; scale bars = 20 μm

Confocal microscopy indicated that epidermal keratinocytes and dermal cells, mainly fibroblasts, are the most common cells to express CBRs (Figures 2 and 3), whereas WB analysis additionally confirmed that CBRs occurred in the whole skin tissue samples (Figure 4). For further verification of whether CBRs are expressed by both or other cell types, we established an enzymatic‐based protocol for skin‐derived primary cell cultures. After enzymatic separation of the epidermis from the dermis and further enzymatic disruption of both compartments, we initiated primary cell expansion separately. Specific culture conditions were applied for epidermal keratinocytes, using commercially available media with well‐defined and selective culture conditions. With respect to the dermis, where in‐situ confocal microscopy analysis shows that, in addition to fibroblasts, other dermal cells also show strong CBRs expression (eg, hair follicles, sebaceous glands, sweat glands), we expanded obtained dermal cell suspensions in basic culture conditions, resulting in the maintenance of all different cells. After >1 week, primary colony‐forming units were visible, and after another 2 weeks, confluent cells were obtained. Although some heterogeneity in the keratinocyte cultures was observed, resulting from residual fibroblast contamination, the application of trypsin‐EDTA solution at different detachment times was used. It successfully removed fibroblasts, which, in contrast to epithelial cells, are detached more rapidly, allowing us to increase keratinocyte homogeneity. Subsequently, we simultaneously performed morphological and immunophenotypic cell analysis with CBRs expression. We used triple confocal microscopic staining, applying CBR antibodies with phalloidin staining to visualize the intracellular actin architecture and cell shapes (Figure 5). Based on phalloidin staining, keratinocytes showed more oval morphology and a slightly weaker reaction for actin content in contrast to fibroblasts (Figure 5B,J). Fibroblasts not only exhibited a rich actin cytoskeleton, but also demonstrated an elongated morphology (Figure 5F,N). Regarding CBRs expression, keratinocytes and fibroblasts demonstrated cellular expression patterns similar to CBR1 and CBR2. Antibodies showed concentrated expression in cell membranes, cytoplasm, and nucleus, respectively. In all cases, however, CB2 expression seemed to be higher (Figure 5I, M) compared to CBR1 (Figure 5A,D), as confirmed by fluorescence intensity analysis (data not shown).

**FIGURE 6:**
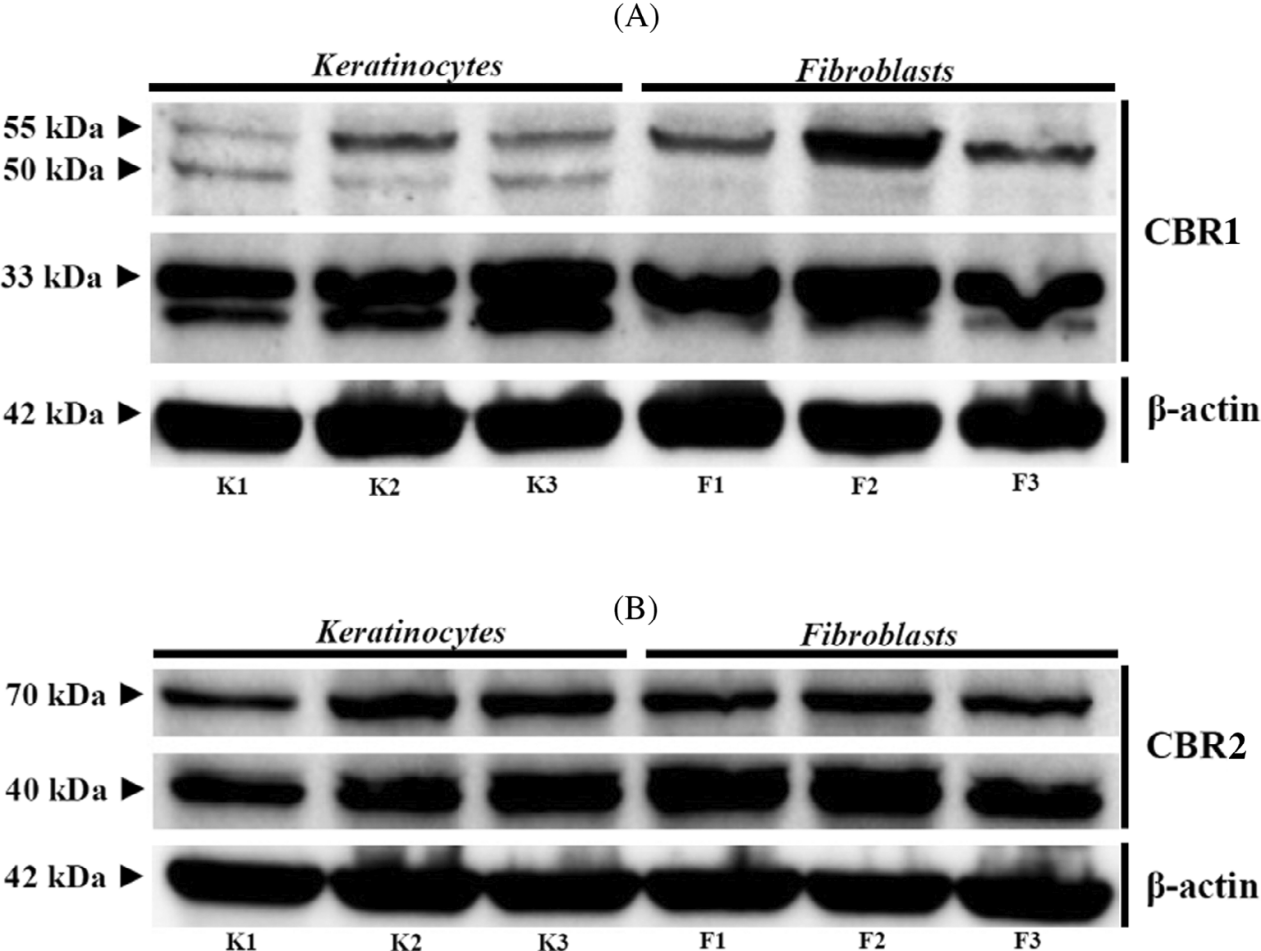
Western‐blot analysis of CBR1 (A) and CBR2 (B) expression in 3 different primary skin‐derived keratinocytes (K1‐3) and fibroblasts (F1‐3). The β‐actin was used as reference protein

Although WB analysis in whole‐tissue samples confirmed the presence of CBRs, we further verified their expression in primary equine keratinocytes and fibroblasts (Figure 6). The WB for the cellular expression of CBR1 showed results that differed slightly from those obtained using skin biopsy samples. For CBR1, we observed 2 immunoreactive bands, 55 kDa, visible in keratinocytes and fibroblasts, as well as the less visible 50 kDa band in keratinocytes, completely absent in fibroblasts. In turn, strong reactivity for the 33 kDa band was noted in lysates originating from keratinocytes and fibroblasts (Figure 6A). Regarding CBR2, WB assays showed 2 immunoreactive bands approximately at 70 kDa and a lower‐molecular‐mass form at approximately 40 kDa, each in a line corresponding to the reaction observed in skin biopsy samples (Figure 6B). The β‐actin immunoreactivity as reference protein was also correctly observed in keratinocytes and fibroblasts, with a band corresponding to 42 kDa. One immunoreactive band in each lane corresponded to equine skin biopsy samples from 15 different individuals, with a molecular mass of 50 kDa (Figure 4). In the WB assay for CBR2 in skin biopsy samples, 2 bands were detected at approximately 70 and 40 kDa (Figure 4B). The lower band corresponded to a deglycosylation or proteolysis product of the 70 kDa band. The lane on the right shows the presence of similar bands in samples of equine cerebral cortex protein extract, which was used as a positive control for both CBR1 and CBR2 (Figure 4).

**FIGURE 7:**
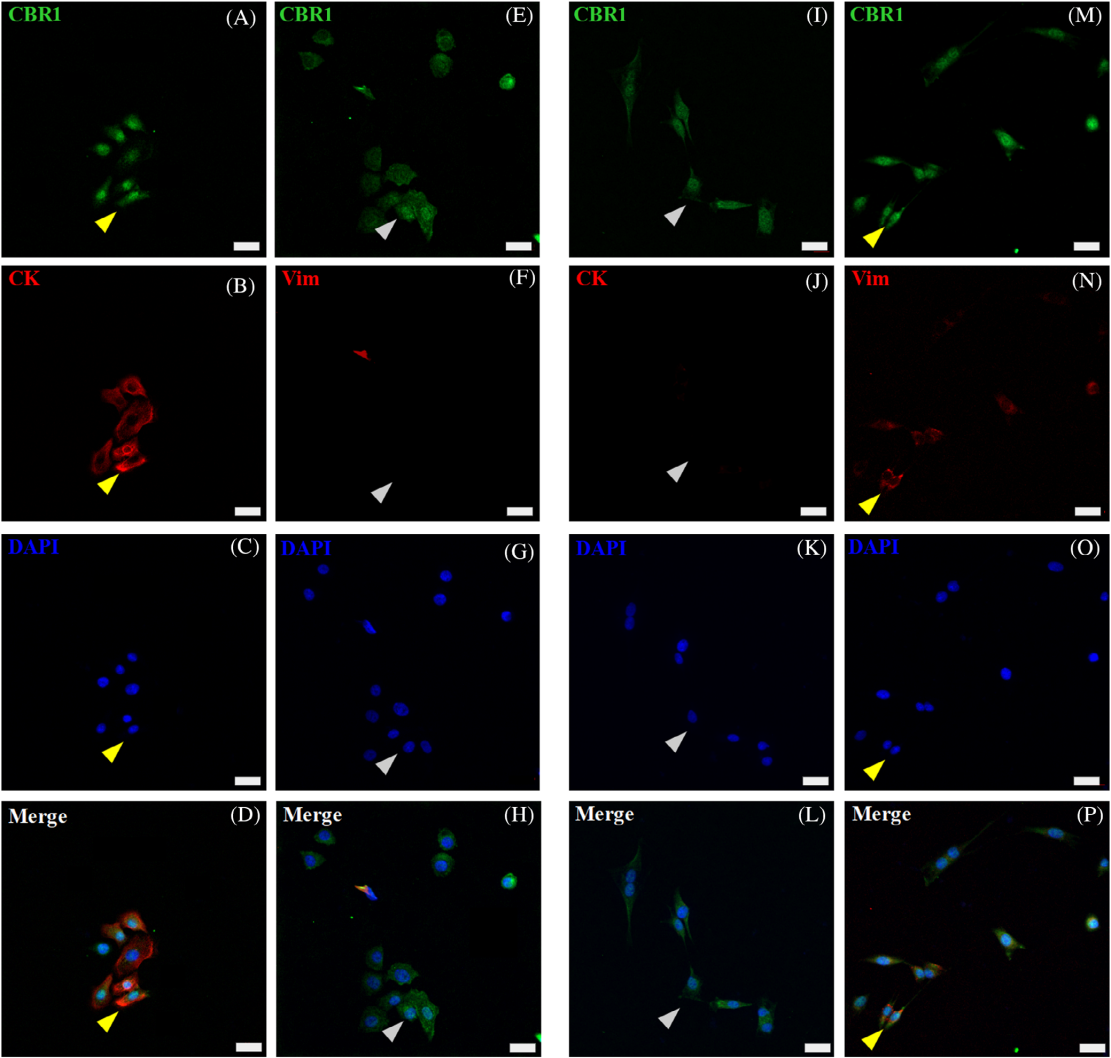
CBR1 (CBR1‐green) confocal microscopic results, showing images of equine primary skin‐derived cultures of keratinocytes (A‐H) and fibroblasts (I‐P). CBR1 immunoreactivity in keratinocytes (A and E) and fibroblasts (I and M). For keratinocyte and fibroblast immunophenotyping, co‐labeling of CBR1 with specific epithelial pan‐cytokeratin (CK‐red) (B) and fibroblast vimentin (Vim‐red) (N) markers was performed (yellow arrows indicate double positive cells). Inverse staining of Vim‐red on keratinocytes (F) and CK‐red on fibroblast (J) with CBR1 was additionally performed to distinguish both primary cell types (gray arrows indicate single positive cells). The DAPI (blue) was used for cell nucleus counterstaining; images were taken under 40x magnification; scale bars = 20 μm

**FIGURE 8:**
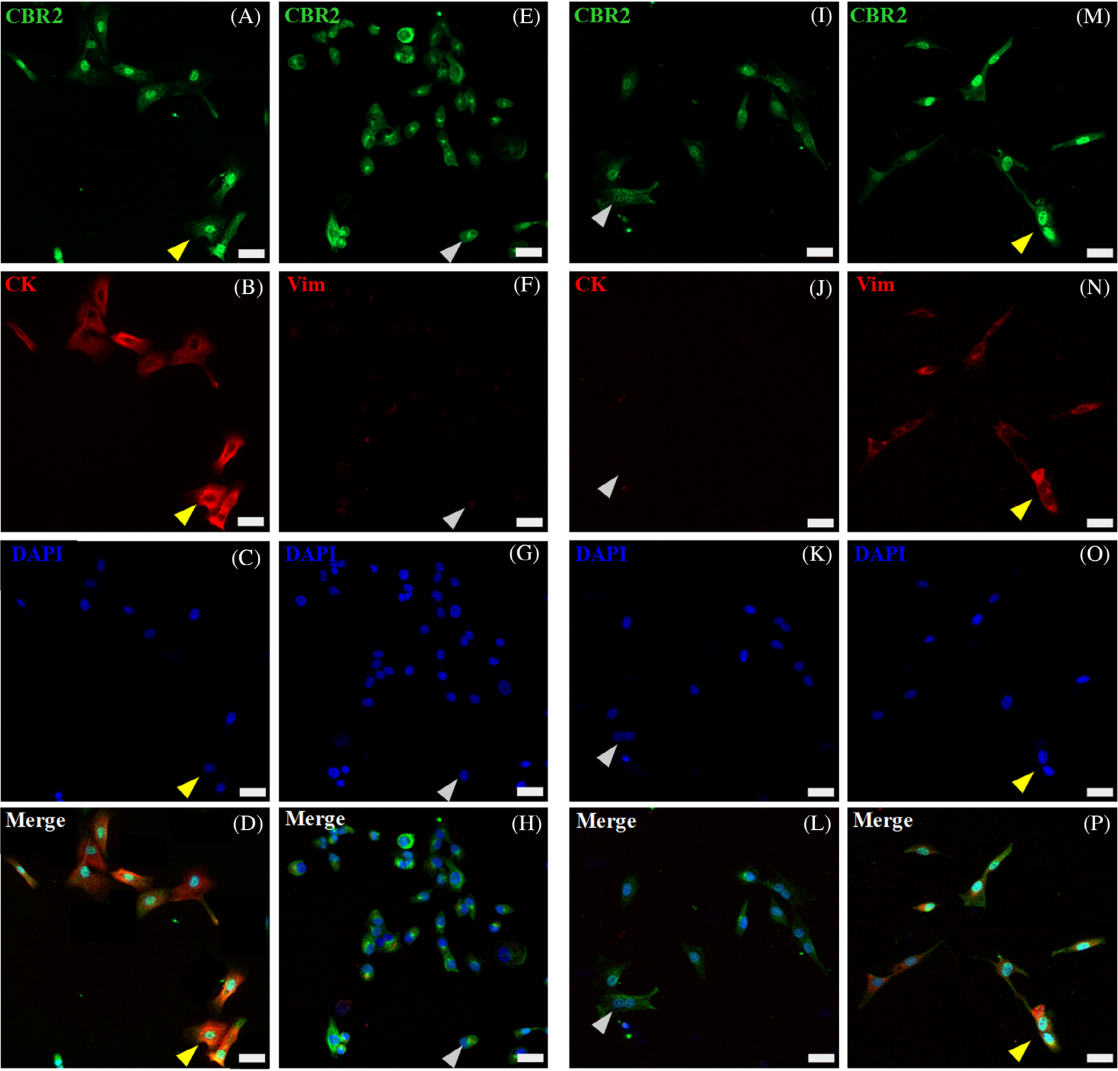
CBR2 (CBR2‐green) confocal microscopic results, showing images of equine primary skin‐derived cultures of keratinocytes (A‐H) and fibroblasts (I‐P). CBR2 immunoreactivity in keratinocytes (A and E) and fibroblasts (I and M). For keratinocyte and fibroblast immunophenotyping, co‐labeling with specific epithelial pan‐cytokeratin (CK‐red) (B) and fibroblast vimentin (Vim‐red) (N) markers was performed (yellow arrows indicate double positive cells). In turn, inverse staining of Vim‐red on keratinocytes (F) and CK‐red on fibroblast (J) was additionally performed to distinguish both primary cell types (gray arrows indicate single positive cells). The DAPI (blue) was used for cell nucleus counterstaining; images were taken under 40x magnification; scale bars = 20 μm

Using pan‐cytokeratin (pan‐CK) antibody recognizing the acidic and basic (types I and II) sets of cytokeratins 1‐8, 10, 14, 16, and 19, we specifically co‐immunolabeled keratinocytes cultures with CBRs (Figures 7A‐D and 8A‐D). Keratinocytes showed high Pan‐CK expression, but its detectable levels also were observed in single primary skin dermal‐derived cells, suggesting that the culture conditions used are appropriate for other cells of epithelial origin, such as hair follicle cells. In situ analysis of whole skin tissue using the Pan‐CK marker confirmed its high expression and common localization in regions where high CBR2 and substantially less CBR1 immunoreact was observed (data not shown). In vitro analysis with co‐labeling of Pan‐CK (red) with CBR1 and CBR2 confirmed the expression of receptors in the skin epithelia (Figures 7A‐D and 8A‐D). In turn, vimentin, considered a fibroblast marker, was expressed at low levels by keratinocytes (Figures 7E‐H and 8E‐H), but significantly overexpressed by primary dermal‐derived cells (Figures 7M‐P and 8M‐P). In contrast, co‐labeling cells with pan‐CK marker verified the epithelial or non‐epithelial origin of dermal cells, which all demonstrated a similar expression of CBRs (Figures 7I‐L and 8I‐L).

### 3.6 Gene expression analysis

**FIGURE 9:**
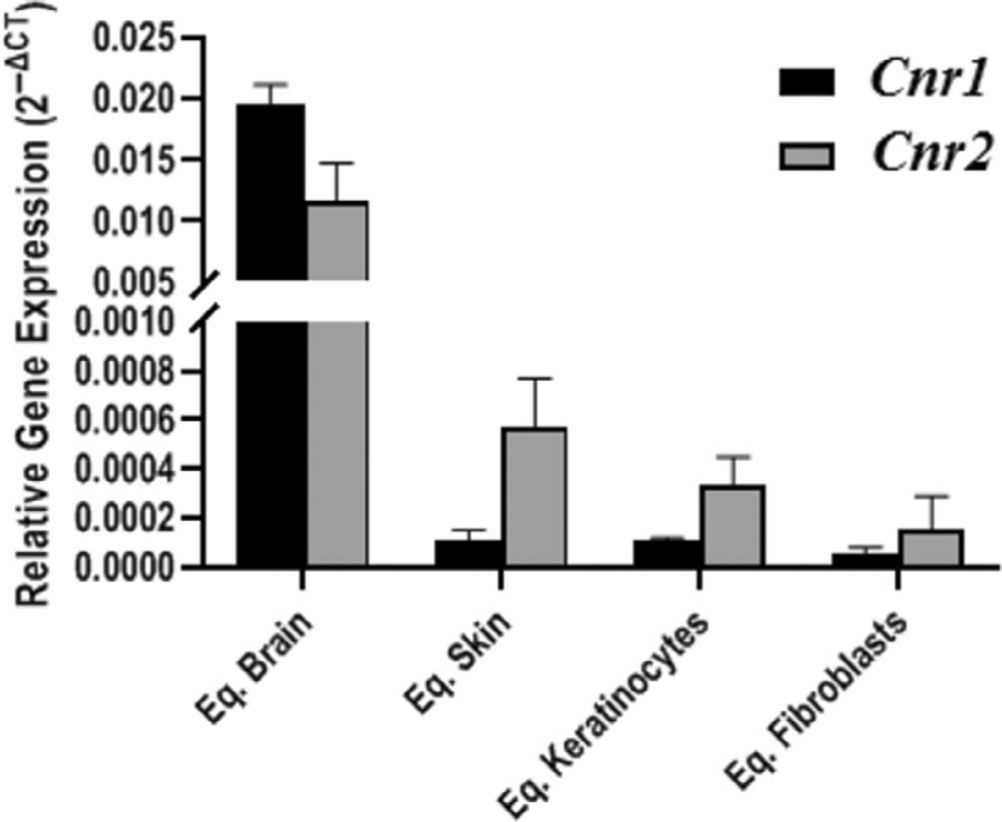
Comparison of the relative expression level of *Cnr1* and *Cnr2* in equine brain (Eq. Brain); skin (Eq. Skin); keratinocytes (Eq. Keratinocytes) and fibroblasts (Eq. Fibroblasts). The transcript level in each biological sample is displayed as the mean value of 2^−ΔCT^ ± SEM

We introduced 3 common equine housekeeping genes (HKGs) for internal controls, namely glyceraldehydes 3‐phosphate dehydrogenase (*Gapdh*), actin‐β (*Actb*), and β2‐microglobulin (*β2m*) (Table 2). For target genes, we projected 2 primer pairs recognizing 2 different transcript regions for *Cnr1* and *Cnr2*, respectively. Gene detection and expression analyses were performed using real‐time PCR with signal detection, applying a common fluorescent dye (data not shown). Subsequently, after selecting the most efficient primer pairs, we performed final gene expression analysis using primer pairs amplified 66 nucleotide sequence for *Cnr1* and 89 nucleotide sequence for *Cnr2*. Absolute verification of the target transcripts was performed using 6‐carboxylfluorescein 6‐FAM‐labeled universal probe library (UPL) probes hybridizing between the flanking forward and reverse primer sequences. Data concerning relative mRNA expression levels of the investigated genes are presented in Figure 9. The transcript level in each biological sample is displayed as the mean value of 2^−ΔCT^ ± SEM.

**TABLE 2:** Equine forward and reverse sequences for target *Cnr1* and *Cnr2* genes and HKGs with their respective FAM‐labeled probes

Gene	Primer sequences: forward (F: 5′‐3′) and reverse (R: 3′‐5′)	UPL probes	NCBI accession number
Target genes			
*Cnr1*	F: ATCCTAGATGGCCTTGCAGA R: TGAGCCCACATAGAGCAGGT	UPL9	NM_001257151.1
*Cnr2*	F: CCCAAAAGATAGCTATTGCAGTG R: GGCCAGGATGAGATAGAGCA	UPL80	NM_001257179.1
Housekeeping genes (HKGs)			
*Actb*	F: CTCCATTCTGGCCTCATTGT R: GTCGTACTCCTGCTTGCTGA	UPL11	NM_001081838.1
*Gapdh*	F: AAGCTTGTCATCAACGGAAAG R: TTGATGTTGGCGGGATCT	UPL9	NM_001163856.1
*β2m*	F: TGTTCCGAAGGTTCAGGTTT R: CAGGAAATTTGGCTTTCCATT	UPL3	NM_001082502.3

Our analysis indicated the presence of both *Cnr1* and *Cnr2* in equine brain, skin, keratinocytes, and fibroblasts. We noticed the significantly higher relative expression of CBRs mRNA in brain tissue for both genes compared to the remaining analyzed material, verified using the Kruskal‐Wallis ANOVA test (*P*‐value = .007 for *Cnr1*, *P*‐value = .0005 for *Cnr2*), and results including significant differences are summarized in Table 3.

**TABLE 3:** Statistical analysis of *Cnr1* and *Cnr2* expression level in different biological samples (equine brain, skin, keratinocytes, and fibroblasts)

Gene	Biological Material				Kruskal‐Wallis ANOVA
	Brain	Skin	Keratinocytes	Fibroblasts	
	Relative gene expression (2^−ΔCT^ ± SD)				
*Cnr1*	1.96E−02 ± 1.53E−03	1.14E−04 ± 4.17E−05	1.09E−04 ± 1.06E−05	5.40E−05 ± 3.06E−05	*P* = .007*
*Cnr2*	1.16E−02 ± 3.11E−03	5.75E−04 ± 1.96E−04	3.25E−04 ± 1.16E−04	1.52E−04 ± 1.40E−04	*P* = .0005*

*Note*: Data are presented as the mean value of 2^−ΔCT^ ± SD; *P*‐value <0.05 was considered statistically significant and marked with asterisk (*).

Although the transcript levels of *Cnr1* and *Cnr2* in skin, fibroblasts, and keratinocytes were low, they were still detectable, confirming mRNA presence of the investigated genes in those samples.

## 4 DISCUSSION

The animal ECS has been identified in all vertebrates and many in‐vertebrate species, where it participates in the regulation of many biological mechanisms.^5^ The tissue distribution and expression patterns of CBRs are comparable and well characterized in several mammalian species, but they still need to be investigated in equine tissues.^7^ Our strategy to identify CBRs in the equine cutaneous system has its roots in observations in human medicine and basic science investigations, where in vivo and in vitro approaches clearly show that CBRs are an exciting target for dermatological treatments in humans.^13^, ^33^ Moreover, considering their utilization and promising results as a therapeutic target in many medical aspects, their application in equine veterinary medicine also is of great interest.^5^, ^33^, ^34^ In that field, treatments, which are still under development, are highly promising because their main advantage is the limited interaction of administrated cannabinoid‐based compounds with the CNS. Unfortunately, many CBRs‐targeted drugs cross the blood‐brain barrier (BBB), resulting in many undesired adverse effects.^15^

As in humans, expression of CBR1 and CBR2 in the normal skin of animals is balanced and controls cutaneous homeostasis.^13^, ^35^ Several studies show that the application of different cannabinoid‐based ligands influences CBRs expression. Exogenous application of AEA significantly influences CBR1‐expressing keratinocytes in situ as well as in the skin‐derived cells via the downregulation of keratins 6 and 16 and inhibition of epidermal keratinocyte proliferation.^36^ The endogenous release of AEA inhibits protein kinase C activity in a CBR1‐dependent manner and modulates keratinocyte differentiation programs.^16^ In mice with CBR1 keratinocyte‐specific deletion, the experimental induction of contact hypersensitivity response occurs with significantly higher intensity than normal stable skin expressed CBR1.

Moreover, receptor deletion in keratinocytes results in the amplified secretion of C‐X‐C motif chemokine ligand 10 (CXCL10) and C‐C motif chemokine ligand 8 (CCL8), key chemokines responsible for myeloid cell infiltration.^17^ In turn, their infiltration may up‐regulate CBR2, which has been confirmed in an experimental skin model of wound healing.^37^ Using animal models, it was shown that neutrophil activity is increased in CBR2^−/−^ mice, and under normal conditions, CBR2‐dependent recruitment is impaired after agonist treatment.^38^ In another study, CBR2 attenuated the inflammatory response in a skin wound healing model, and the external application of dimethylbutyl‐deoxy‐Delta‐8‐THC (JWH133) or *N*‐(Piperidin‐1‐yl)‐1‐(2,4‐dichlorophenyl)‐1,4‐dihydro‐6‐methylindeno [1,2‐*c*]pyrazole‐3‐carboxamide (GP1a), CBR2 agonists, decreased the proinflammatory injury response in contrast to animals treated with 6‐Iodo‐2‐methyl‐1‐[2‐(4‐morpholinyl)ethyl]‐1H‐indol‐3‐yl](4‐methoxyphenyl)methanone (AM630), CBR2‐antagonist.^39^

In veterinary medicine, CBRs expression analysis has been performed in the peripheral nervous system (PNS) and skin of animals, with detailed characterization in dogs and cats. In both cases, proinflammatory skin dermatoses significantly impaired CBRs expression and distribution.^20^, ^23^, ^24^, ^40^, ^41^ Regarding horses, gene and protein identification of CBR1 and CBR2 expression in whole skin tissue and primary skin‐derived epidermal keratinocytes and dermal cells has never been evaluated. However, studies have attempted to identify CBRs in the skin epithelia in humans. Confocal microscopy results demonstrate the distribution of CBRs with complementary expression patterns, mostly in epidermal keratinocytes and hair follicles, and other human skin resident immune cells. Moreover, CBRs were present on DRG nerve endings, with particular expression on afferent calcitonin gene‐related peptide (CGRP)‐positive peptidergic nerves.^42^ In the normal skin of dogs and cats, CBRs tissue expression patterns are comparable and concern DRG terminals and skin cells, in agreement with previous findings.^23^, ^24^ The expression and distribution of CBRs along DRG neurons, the longest axonal DRG projections in vertebrates, presents therapeutic advantages. The DRG axons terminate in most epithelial tissues including the skin and are responsible for transmitting sensory‐like signals where axonal transport plays an important role.^43^, ^44^ One study confirmed CBRs expression along the DRG sensory neurons.^25^ Results of that study were consistent with previous observations in other species and those presented by other groups.^21^, ^22^, ^45^, ^46^ The DRG nerve terminals play a specific role in CBRs neurobiology and have been investigated in many aspects, mainly neuroinflammation.^47^ Studies on rat peripheral tissues show that experimental sciatic nerve injury is an inducible factor for detecting CBRs at mRNA and protein levels.^44^, ^48^ There is strong evidence that the peripheral expression of CBRs might result from DRG sensory neuron‐dependent axonal flow, and their delivery from CNS or production in situ is highly regulated by skin conditions.^49^ We observed that detecting CBRs transcripts by real‐time PCR was low in whole skin samples, whereas levels in primary keratinocyte and fibroblast cultures were even lower, albeit with acceptable detection. At the same time, all gene expression analyses in the whole‐skin and primary cultures were performed in comparison to brain reference material, where expression was almost 200‐fold higher. Moreover, the molecular biology of CBRs transcript expression and regulation seems to be more complex and dynamic. The transcript cargo along DRG neurons depends on neurotransmission conditions, its short half‐life, and several epigenetic factors, which should be considered with regard to CBRs mRNA regulation and expression.^50^, ^51^ These unusual expression patterns of *Cnr1* and *Cnr2* transcripts in the skin seem reliable and have excellent primer efficiency for target genes. Three common HKGs used in our experiments provided positive amplification results. In contrast, protein expression can be more precisely captured because of their longer exposure through synaptic terminals and target cell membranes, including keratinocytes.^37^, ^48^, ^52^ Therefore, we decided to use PGP 9.5, a pan neuroendocrine marker mapping all peptidergic and non‐peptidergic DRG axonal nerve terminals.^29^, ^53^ Biochemically, PGP 9.5 is ubiquitin terminal carboxyl hydrolase L1 (UCHL1), a deubiquitinating enzyme of the ubiquitin‐protesome system (UPS), a largely abundant protein of the CNS and PNS.^54^, ^55^ It regulates several neurobiological mechanisms such as axonal transport, neuronal guidance, and synaptic functions.^55^, ^56^, ^57^ Moreover, PGP 9.5 participates in regulating neural crest cell precursors and their repopulation to peripheral epithelia. In postnatal skin, PGP 9.5 is still expressed and led to identification of an epithelial neuro‐immuno‐endocrine cell situated in the basal layers of epidermis. For that reason, PGP 9.5 serves as an excellent connecting marker between neuronal and non‐neuronal cells.^29^, ^57^, ^58^, ^59^ Our studies observed heterogeneous PGP 9.5 immunoreactivity in different skin areas, but 2 main staining patterns correspond to cytoplasmic and nerve fiber‐like structures. Cytoplasmic expression detects neuro‐immune‐endocrine cells of the epidermis and single cells within all dermal compartments, mainly of the inner root sheath of hair follicle cells, sebaceous glands, and sweat glands. In turn, single nerve terminals were detected almost across the entire skin. In normal skin, epidermal and dermal PGP 9.5 nerve terminals are rare, and their low quantities detected in our studies were consistent with studies on human and equine skin as well as horse intestine.^29^, ^53^, ^59^ In contrast to tissue distribution in equine cells and tissues, antibody specificity using WB needs to be included, especially where analyzed target proteins belong to the large superfamily of GCPRs.^60^ The anti‐CBR1 antibody provided a single 50 kDa band in the whole equine skin and brain lysates, and those results correspond with those obtained in human endothelial cells, immune cells in mice, and macaque brain tissue.^6^, ^61^ Interestingly, less intensive bands observed around 50 kDa and, at approximately 33 kDa are probably consistent with glycosylated and non‐glycosylated CBR1 forms.^62^, ^63^ In Human Melanoma Cell Line (SK‐mel‐1) extracts, CBR1 had only a band with a molecular mass of 37 kDa, whereas the reactivity for anti‐CB2 antibody had 2 bands of approximately 40 and 70 kDa, respectively.^64^ Several reports indicate the presence of a glycosylated form of CBR2 at approximately 46 kDa and a non‐glycosylated form of CBR2 at approximately 41 kDa, which might explain the detection of the 40 kDa band in our studies.^65^, ^66^, ^67^ The CBR2 post‐translational modification has not been reported, but we suspected that the multiband we found might reflect glycosylated forms.^68^, ^69^ Only 1 study reported WB for CBRs using equine source tissue, namely the cervical DRG at high molecular masses for CBR1 (100 and 120 kDa) and 90 kDa for CBR2. Similar results for keratinocytes have been reported for normal human epidermal keratinocytes (NHEK) and a human spontaneously immortalized keratinocytes (HaCaT): approximately 50 kDa CBR1 and 46 kDa (or 60 kDa).^16^, ^70^ The immunogene peptides for equine and human CBR1 and CBR2 are summarized in a Table 4. A number of factors may affect the molecular mass of proteins found in the WB reactions, such as a source of the protein (tissue or cellular lysate origin) animal species, and producer of antibodies.

**TABLE 4:** Amino acid sequences for equine (Eq) and human (Hu) immunogenes with peptide length and identity, recognizing CBR1 and CBR2 proteins

Protein name	Amino acid (aa) length	Aa sequences for equine (Eq) and human (Hu) immunogene peptides	Peptide identity	Protein accession number
Eq.CBR1	99	MKSILDGLADTTFRTITTDLLYVGSNDIQYEDIKGDMASKLGYFPQKFPLTSFRGSPFQEKMTAGD **S** PQLVPADQVNITEFYNKSLSS **Y** KENEENIQCG	97.98%	NP_001832.1
Hu.CBR1		MKSILDGLADTTFRTITTDLLYVGSNDIQYEDIKGDMASKLGYFPQKFPLTSFRGSPFQEKMTAGD **N** PQLVPADQVNITEFYNKSLSS **F** KENEENIQCG		NP_001153698.1
Eq.CBR2	33	ME **R** CWVTE **A** ANGS **T** DGLD **F** NPMKDYMILS **SS** QK	81.82%	NP_001244108.1
Hu.CBR2		ME **E** CWVTE **I** ANGS **K** DGLD **S** NPMKDYMILS **GP** QK		NP_001832.1

*Note*: Accession numbers for all peptides are included with differences in amino acid sequences (bold positions).

Our study had some limitations. Because samples were obtained from horses at euthanasia, our study group was not homogeneous, influencing our final results. At the same time, skin biopsy samples were obtained from only 1 side of the body, without comparative analysis of other regions. Therefore, it is necessary to conduct further research to assess the distribution of CBRs elsewhere in the body.

## 5 CONCLUSIONS

Our study provided detailed gene and protein expression of CBRs in whole equine skin tissue and its respective primary cells. The equine CBR1 and CBR2 are stably expressed by keratinocytes, fibroblasts other dermal cells, and DRG terminals. Applications of CBR ligand compounds were strongly prohibited for a long time. The detailed crystal structural organization of CBRs led to an explanation of molecular interactions of ligand recognition with receptor pocket binding sides, shedding new light on drug candidates.^71^, ^72^ This finding has led to the exploration and development of a new generation of cannabinoid based drugs with minimal interactions with CNS and promising therapeutic effects.^73^ The results presented here provide the basis for a new and interesting therapeutic target for the development and translation of peripheral treatments for clinical dermatology in equine veterinary medicine.

## CONFLICT OF INTEREST DECLARATION

Authors declare no conflict of interest.

## OFF‐LABEL ANTIMICROBIAL DECLARATION

Authors declare no off‐label use of antimicrobials.

## INSTITUTIONAL ANIMAL CARE AND USE COMMITTEE (IACUC) OR OTHER APPROVAL DECLARATION

Dermatological examination performed in this study was non‐invasive and is routinely performed in everyday medical practice. The equine tissue samples used in the study were obtained from an abattoir of healthy horses slaughtered for consumption. Ethical approval from the local animal health commission was not required.

## HUMAN ETHICS APPROVAL DECLARATION

Authors declare human ethics approval was not needed for this study.

## Supporting information


**Data S1.** Supplementary material.

**Figure S1.** Triple confocal microscopic in‐situ expression of CBR1 (A‐D) and CBR2 (E‐H), PGP 9.5 (B and F) and DAPI (C and G) in the whole brain cortex tissue.

**Table S1.** Characteristics of the horses and their respective skin samples (S1‐S15).
