# Supplementary material for: The cannabinoid receptors system in horses: Tissue distribution and cellular identification in skin
Source: J Vet Intern Med. 2022 Jul 8;36(4):1508–24. doi: 10.1111/jvim.16467 (PMC9308437; doi:10.1111/jvim.16467)
Supplement: Supplementary file 1 — Data S1. Supplementary material. [file JVIM-36-1508-s001.zip › Appendix supplementary.pdf]

Supplementary Data

Animals and Sample collection

Only healthy horses slaughtered for consumption were included in the study group (n = 15), with ages ranging from 2 to 25 years (mean: 13.44; SD ± 7.15). All data are summarised in Table 5.

The clinical condition of the horse's skin was assessed as normal based on a dermatological examination performed before slaughter. After slaughter, the thoracic limbs of the horses were amputated at the carpus level. The skin from the dorsal part of the metacarpus was selected for a 5-mm punch skin biopsy (Kai Medical, Solingen, Germany), with an area of about 5 x 5 cm. Subsequently, it was scrubbed with chlorhexidine (Impag Chemicals, Warsaw, Poland) for about 2 minutes, and the central part of the clipped area was shaved with a razor and repeatedly rubbed with compresses soaked in 70% isopropyl alcohol solution for about 60 seconds. Three full-thickness skin pieces 5 mm in diameter were cut under sterile conditions with separate biopsy punches for each individual from each horse (S1–15) and rinsed in 70% ethanol to remove residual blood and other contaminations; subsequently, they were transferred into stabilisation solutions depending on the laboratory purpose described below. From the moment of slaughter, it took no more than 10 minutes to obtain the material for testing. In addition, 5-mm-diameter cerebral cortex samples (B1–3) were collected from three other horses as reference material.

Tissue processing

For histology, formalin-fixed paraffin-embedded (FFPE) blocks were prepared. Briefly, skin biopsies were placed into the fixative solution of 10% buffered formalin for 48 hours and subjected to the routine protocol for tissue processing, dehydration in increasing ethanol solution, two incubation in xylene and paraffin embedding.

For confocal microscopy, the skin biopsies were rinsed in 70% ethanol and immediately placed in a freshly prepared ice-cold fixative solution overnight, which contained 4% paraformaldehyde (PFA, POCH S.A., Gliwice, Poland) and 0.02% saturated picric acid (POCH S.A., Gliwice, Poland) prepared in 0.133 mM of Sörensen buffer, pH = 7.4. Subsequently, the biopsies were kept into 15% sucrose solution in phosphate buffer saline (PBS) for 24 hours and further incubated for 24 hours in 30% sucrose (POCH S.A., Gliwice, Poland) solution with 10% glycerol (v/v) (POCH S.A., Gliwice, Poland). After this, they were immersed in an OCT medium and stored at -80°C.

For the initiation of primary cell cultures of keratinocytes and fibroblasts, the skin biopsies were immediately placed in the DMEM-F12 media supplemented with 10% foetal bovine serum (FBS, Gibco-Thermo Fisher Scientific, Waltham, USA) and 3% antibiotic solution (Sigma-Aldrich, Saint Louis, USA) and transported to the laboratory.

For molecular biology analysis, skin biopsies were immediately placed in a stabilisation reagent, *RNA-Stay* (A&A Biotechnology, Gdansk, Poland), at 4°C on ice. Soon after delivery to the laboratory, biopsies were transferred to -80°C and used in the gene expression analysis, applying real-time PCR and protein detection in Western blot.

Histological staining

From FFPE, 5-µm paraffin sections were prepared using a microtome instrument and subjected to standard deparaffinisation. Each sample was stained by H&E with Delafield haematoxylin (Roth GmbH, Karlsruhe, Germany) and eosin (POCH S.A., Gliwice, Poland). The slides were examined and photographed under a light microscope (Nikon Eclipse 80i; Nikon, Melville, NY, USA) with a video camera using the NIS-Elements AR 2.30 (Nikon) imaging software.

Confocal microscopy and image analysis

The OCT-embedded skin biopsies were removed from a low-temperature freezer and transferred into a cryostat microtome (Leica, Wetzlar, Germany) instrument under -24°C. The 12-µm-thick frozen sections were prepared for two-dimensional visualisation of proteins onto poly-L-lysine glass slides (Superfrost Ultra Plus, Thermo Fisher Scientific, Waltham, USA), dried at room temperature overnight and subjected to the immunofluorescence protocol, with minor modifications as described elsewhere [45]. Briefly, sections were incubated in post-fixative solution containing 10% DMSO (Sigma-Aldrich, Saint Louis, USA), 2.5% glacial acetic acid (POCH S.A., Gliwice, Poland), 0.5% glycerol (POCH S.A., Gliwice, Poland), 0.05% Triton X-100 (Sigma-Aldrich, Saint Louis, USA) and 0.05% Tween 20 (Sigma-Aldrich, Saint Louis, USA) prepared in 70% ethanol in PBS and incubated at 37°C for 45 min. Subsequently, the section was hydrated in PBS for 3 x 5 min at room temperature (RT), and blocking solution (BS) containing 5% bovine serum albumin (BSA) (Lab Empire, Rzeszow, Poland), 3% normal goat serum (NGS) (Abcam, Cambridge, Great Britain), 0.05% Triton X-100 and 0.05% Tween 20 was prepared in PBS, followed by incubation on slides for 2 hours at RT. After rising 3 x 5 min in PBS, the different sets of primary

antibodies, namely rabbit polyclonal anti-CBR_1 (1:250, Thermo Fisher Scientific, Waltham, USA) and rabbit polyclonal anti-CBR_2 (1:250, Thermo Fisher Scientific, Waltham, USA) in combination with chicken anti-PGP 9.5 (1:1000, Thermo Fisher Scientific, Waltham, USA), mouse monoclonal anti-pan cytokeratin (1:500, Thermo Fisher Scientific, Waltham, USA) and mouse monoclonal anti-vimentin (1:500, Thermo Fisher Scientific, Waltham, USA) were diluted in BS and incubated overnight at 4^oC. The following day, sections or cell cultures were gently rinsed 3 x 5 min in PBS and incubated with the secondary antibodies goat-anti-rabbit DyLight488, goat-anti chicken DyLight594, and goat-anti mouse DyLight633 (1:500, Thermo Fisher Scientific, Waltham, USA) for 2 hours at RT. Finally, sections were rinsed for 5 x 5 min in PBS and mounted in a ready-to-used medium with DAPI (Santa Cruz Biotechnology, Santa Cruz, USA) for cell nucleus counterstaining. The protocol described above was also used for CBR identification during *in vitro* expansion. All primary and secondary antibodies used in confocal microscopy are summarised in Table 2.

Imaging was performed using a Zeiss Cell Observer SD spinning disk confocal microscope (Carl Zeiss, Jena, Germany) with 20x and 40x objectives. For *in vitro* and *in vivo* analysis, fluorescence detection was achieved by excitation laser lines corresponding to 405 nm for DAPI, 488 nm for CBR1 and CBR2 labeled by DyLight488, and 561 nm for PGP 9.5 labelled with DyLight 594 and 639 nm for vimentin and cytokeratin labelled by DyLight 633. All images were generated with constant camera settings and exposition time. The ZEN software generated the z-stack images (Carl Zeiss, Jena, Germany) and was further processed in the Fiji-ImageJ software (National Institutes of Health, Bethesda, USA). For measuring CBRs protein expression stained by DyeLight488 as fluorescence intensity (FI), the images were converted to the 8-bit greyscale. Next, the application of *Substract Background* and *Filters>Median* algorithms led to a reduced background noise ratio. Finally, *Threshold* function with *Huang* algorithm was applied to exclude residual non-specific signals. The five randomly selected region of interest (ROIs), which contain a group of 10 cells, were chosen with a polygon selection tool. The ROIs were chosen in the whole skin tissue regions marked by white dotted lines (Fig. 2 and Fig. 3) representing the following skin compartments: epidermis, higher and lower regions of the superficial papillary dermis, deeper reticular dermis, and deeper subcutaneous and muscle dermis. For skin dermis, ROIs were extracted as

five randomly selected squares with constant areas just below the analyzed epidermis. Mean FI results are added in supplementary methods in the excel file (Equine.CBRs.FI).

Isolation and cultivation of equine primary keratinocytes and fibroblasts

Briefly, the tissue samples were washed in phosphate-buffered saline (PBS) ("masked for review") containing 1% of streptomycin/penicillin antibiotic solution (Sigma Aldrich, Saint Louis, USA) and kept in dispase II solution (2.4 U/mL, CnT-DNP-10, CellnTec, Bern, Switzerland) at 37˚C for 3 h. After incubation, the epidermis was mechanically separated from the dermis. Dispase II residues were removed by centrifugation.

For the cultivation of keratinocytes, the epidermis was cut into small pieces and incubated in 0.25% trypsin with 0.05% ethyl-enediaminetetraacetic acid (EDTA) (Trypsin-EDTA Solution, Sigma Aldrich, Saint Louis, USA) for 10–15 min at 37˚C. Trypsin was inactivated with the addition of a medium containing 10% foetal bovine serum (FBS, Gibco-Thermo Fisher Scientific, Waltham, USA), and the cells were collected by centrifugation (200 x g, 7 min, 4ºC). The cell pellet was resuspended in a culture medium, and the cells were seeded on a 6-well culture plate. Trypsinisation and centrifugation were repeated with the tissue leftovers several times to obtain as many cells as possible. The derived cells were cultivated in Epidermal Keratinocyte Medium (CnT-09, CellnTec, Bern, Switzerland) supplemented with CnT-IsoBoost Supplement (CnT-ISO-50, CellnTec, Bern, Switzerland) and 1% of streptomycin/penicillin solution (Sigma Aldrich, Saint Louis, USA) at 37˚C and 5% CO_2.

For the cultivation of skin dermal-derived cells, the dermis was cut into small pieces and seeded on a 6-well culture plate in DMEM medium ("masked for review") supplemented with 10% FBS (Gibco-Thermo Fisher Scientific, Waltham, USA), 1% of streptomycin/penicillin solution (Sigma Aldrich, Saint Louis, USA) and 2mM of L-glutamine (Sigma Aldrich, Saint Louis, USA) at 37˚C and 5% CO_2.

As soon as the cells (keratinocytes or dermal cells) reached 80% confluence, they were passaged into successive culture vessels. To avoid the contamination of keratinocyte cultures with other dermal cells, mainly fibroblasts, different detachment times were employed to separate fibroblasts from other epithelial cells (after application of trypsin-EDTA solution, fibroblasts detach faster, which facilitates removal from the keratinocyte culture).

CBR gene and protein expression

The equine CBR expression analysis for their mRNA transcripts and respective proteins were obtained from the Nucleotide Center for Biotechnology Information (NCBI) database.

Both *Cnr1* and *Cnr2* mRNA analyses were performed on predicted sequences from the equine whole-genome sequencing project [43;44]. The sequences were input to the freely available Roche Assay Design Center with the ProbeFinder 2.48 (Roche, Bazylea, Switzerland) software. The two primer projects for *Cnr1* and *Cnr2* and their respective FAM-labeled UPL probes, corresponding to two different regions of each gene, were generated. The primer sequence specificity was further verified using the Nucleotide Basic Local Alignment Search Tool - Nucleotide-BLAST (NCBI, USA). As internal controls, three stable equine housekeeping genes (HKGs), namely *ACTB*, *GAPDH* and *β2M*, were introduced in all gene expression investigations as proposed by Bogaert et al. [49]. Target and HKG sequences are listed in Table 3.

The protein amino acid sequences of N-termini for human CBRs were used as immunogenes to produce CBR antibodies. The extracellular N-termini for both CBRs consisted of a conservative amino sequence peptide within mammalian species. Considering the manufacturers' information, we decided to compare immunopeptide sequences in a set of commercially available antibodies. The most significant similarities in nucleotide sequences resulted in higher cross-reactivity between humans and equines, which was an additional factor indicating the choice of antibody supplier. Their identity to equine sequences was analysed and is summarised in Table 5. The equine endogenous protein control was β-actin.

RNA isolation and reverse transcription

The total RNA from equine skin biopsies as well as primary keratinocyte and fibroblast cells were isolated as described elsewhere [45;46]. Briefly, skin biopsies were transferred from a freezer (-80°C) under laminar flow; after thawing, they were rinsed thoroughly several times in molecular biology water (EurX - Molecular Biology Products, Gdansk, Poland) to remove residual RNA Stay reagent. Subsequently, using sterile tweezers and scissors, the skin was cut into small pieces and placed into a new Eppendorf tube containing 0.5 ml Phenosol Plus (A&A Biotechnology, Gdansk, Poland) on ice. Tissue fragments were homogenised (1500 rpm) using a tissue homogeniser (Pro200, ProScientific, USA), and subsequently, another 0.3 ml of Phenosol Plus was added. For RNA extraction from the primary keratinocytes and fibroblasts, 0.5 ml of Phenosol Plus was used per 6-well plate soon after the cells reached 90% confluence. The cell suspension was further processed as described for the tissue homogenates and shaken on a thermoblock at 1400 rpm, 45 min, 4ºC (Eppendorf, Hamburg, Germany). After the addition of chloroform (POCH S.A., Gliwice, Poland) and centrifugation (12,500 x g, 15 min, 4°C), the clear RNA-

rich phase in the upper space of the tube was carefully removed and mixed with isopropanol (POCH S.A., Gliwice, Poland). All volume was transferred to a new homogenisation membrane tube and centrifuged (11,000 x g, 3 min, 4°C) to eliminate residual tissue and other cell debris. The flow-through volume was transferred to a new tube with a binding membrane and centrifuged (11,000 x g, 1 min, 4°C) to eliminate residual genomic DNA (gDNA). The mixture was washed with DN1 buffer step and treated with the DNase I Kit (EurX – Molecular Biology Products, Gdansk, Poland) to eliminate gDNA. After incubation for 15 min at RT, two washing steps with RBW buffer and centrifugation were performed as described above. The binding membranes were transferred to a new collection tube, and RNA/DNA-free water (EurX – Molecular Biology Products, Gdansk, Poland) was added, followed by incubation for 10 min at RT. After that, RNA-rich samples were obtained by centrifugation at 12,500 x g for 3 min, and the quantity and quality scores were estimated using ImplenNanoPhotometer (Implen Inc., Munich, Germany). The cell and tissue samples with absorbance 260/280 ratios ranging between 1.7 and 2.1 were used. Subsequently, 0.3 µg from skin tissue and 1 µg from cell RNA were used for cDNA synthesis applying the smaRT First Strand cDNA Synthesis Kit (EurX – Molecular Biology Products, Gdansk, Poland) according to the manufacturer's manual.

Real-time PCR

Following cDNA synthesis, samples were diluted with RNA-free water. For gene expression analysis, 1 µl of cDNA (10 ng) per reaction was used in a total volume of 10 µl. The reaction mix (per well) also included 5 µl of UPL ProbeMaster (Roche, Bazylea, Switzerland), 0.5 µM of forward and reverse primers (Genomed, Warsaw, Poland), and 0.2 µM of Universal Probe Library (UPL, Roche, Bazylea, Switzerland) hydrolysis probes. Real-time PCR was performed using the LightCycler 480 II (Roche Molecular Systems Inc., Indianapolis, USA) instrument with the following conditions: pre-incubation at 95°C for 10 min, 50 cycles of amplification: 15 s at 95°C for denaturation, 30 s at 58°C for annealing and 10 s at 72°C for elongation, followed by cooling at 40°C for 10 s. All gene expression analyses were performed in triplicates in the three independent experiments, following the Minimum Information for the Publication of Quantitative Real-time PCR Experiments [50].

Western blot

Skin and brain biopsies were cut into small pieces and mechanically homogenised using an electric tissue homogeniser (ProScientific, Oxford, USA) on ice. Keratinocytes and fibroblasts were harvested after culturing. Skin, brain, and cell samples were lysed with RIPA lysis buffer (Sigma-Aldrich, Saint Louis, USA)

with a protease cocktail inhibitor (Sigma-Aldrich, Saint Louis, USA) for 20 min at 4ºC with shaking. After lysate centrifugation (20 minutes at 12,500 x g, 4°C), supernatants were collected into new Eppendorf tubes. Total protein concentration was measured using the bicinchoninic acid assay kit (Thermo Fisher Scientific, Waltham, USA) [48]. Total protein (20 µg) was denatured (5 min in 95ºC) and subjected to SDS-polyacrylamide gel electrophoresis (SDS-PAGE) on 1.0-mm thick gel (4% stacking gel and a 10% resolving gel) in an electrode buffer: 25 mMTris and 192 mM glycine, 0.5% SDS, pH 8.3 (Bio-Rad, Hercules, USA) [51]. Precision Plus Protein Dual Color Standard (Bio-Rad, Hercules, USA) was used as molecular mass standard. The resolved proteins were transferred to an Immobilon P membrane (Merck-Millipore, Burlington, USA) through semi-dry transfer, and the membranes were blocked with 1% casein blocking buffer (Sigma-Aldrich, Saint Louis, USA) at room temperature for 1 hour. After washing, the membranes were incubated overnight at 4ºC with the primary antibodies rabbit polyclonal anti-CBR1 (1:1000; Thermo Fisher Scientific, Waltham, USA) and rabbit polyclonal anti-CBR2 (1:1000; Thermo Fisher Scientific, Waltham, USA) in phosphate-buffered saline, pH 7.3 (VWR International, Radmor, USA), 0.05% Tween 20 (Thermo Fisher Scientific, Waltham, USA) (PBS-T). Subsequently, the membranes were incubated with HRP-conjugated goat anti-rabbit antibody (1:25000, Invitrogen, Waltham, USA) diluted in PBS-T buffer, and then analysed proteins were visualised with the use of Clarity Western ECL chemiluminescent substrate (Bio-Rad, Hercules, USA) in the ChemiDoc MP Imaging System (Bio-Rad, Hercules, USA). For the loading control, after stripping, membranes were incubated with HRP-conjugated anti-β-actin antibodies (1:5000, Santa Cruz, Santa Cruz, USA) diluted in PBS-T buffer. The obtained Western blot membrane images were analysed with the use of the Image Lab software version 6.0.1 build 34 (Bio-Rad, Hercules, USA).
