# Supplementary figures and images for: The cannabinoid receptors system in horses: Tissue distribution and cellular identification in skin
Source: J Vet Intern Med. 2022 Jul 8;36(4):1508–24. doi: 10.1111/jvim.16467 (PMC9308437; doi:10.1111/jvim.16467)

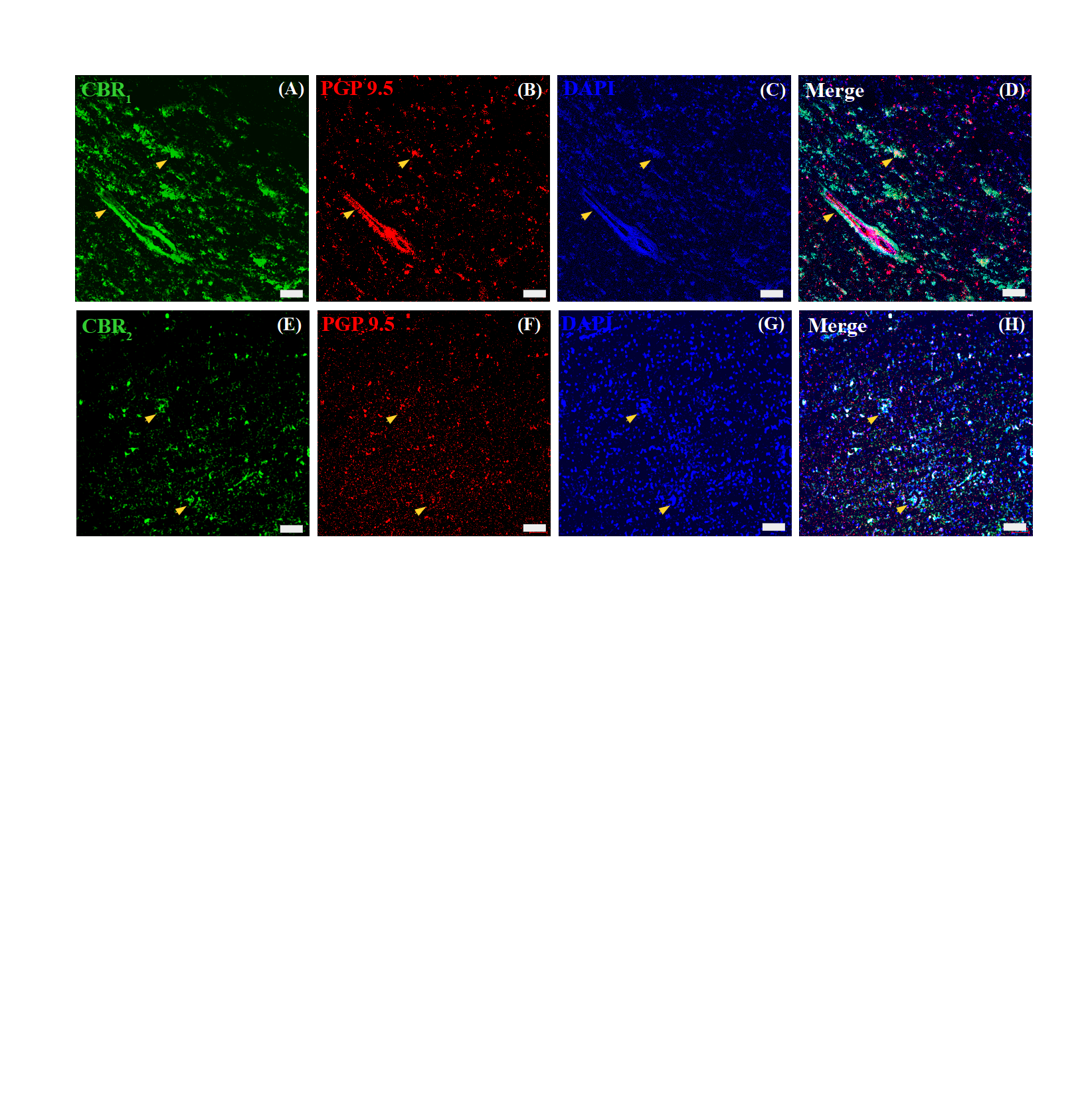

Supplement: Supplementary file 2 — Figure S1. Triple confocal microscopic in‐situ expression of CBR1 (A‐D) and CBR2 (E‐H), PGP 9.5 (B and F) and DAPI (C and G) in the whole brain cortex tissue. [file JVIM-36-1508-s002.tif]
