# Supplementary material for: The cannabinoid receptors system in horses: Tissue distribution and cellular identification in skin
Source: J Vet Intern Med. 2022 Jul 8;36(4):1508–24. doi: 10.1111/jvim.16467 (PMC9308437; doi:10.1111/jvim.16467)
Supplement: Supplementary file 3 — Table S1. Characteristics of the horses and their respective skin samples (S1‐S15). [file JVIM-36-1508-s003.docx]

| Sample ID | Gender | Age (years) | Type | Colour | Pigmentation |
| --- | --- | --- | --- | --- | --- |
| S1 | mare | 13 | warmblood | bay | no |
| S2 | mare | 17 | coldblood | chestnut | no |
| S3 | gelding | 14 | coldblood | chestnut | no |
| S4 | gelding | 5 | warmblood | dark bay | no |
| S5 | mare | 2 | warmblood | bay | no |
| S6 | gelding | 19 | warmblood | chestnut | no |
| S7 | gelding | 6 | warmblood | black | no |
| S8 | mare | 18 | warmblood | bay | no |
| S9 | gelding | 16 | warmblood | chestnut | yes |
| S10 | mare | 19 | warmblood | bay | yes |
| S11 | gelding | 15 | warmblood | dark bay | no |
| S12 | mare | 25 | warmblood | bay | no |
| S13 | mare | 18 | warmblood | dark bay | no |
| S14 | gelding | 12 | warmblood | bay | yes |
| S15 | mare | 22 | warmblood | bay | yes |
| B1 | mare | 3 | coldblood | chestnut | - |
| B2 | gelding | 17 | coldblood | bay | - |
| B3 | gelding | 1 | coldblood | bay | - |

Table 5. Characteristics of the horses and their respective skin samples (S1–S15).
